# Isolated right ventricular hypoplasia associated with cyanotic atrial septal defect: a case report

**DOI:** 10.1093/ehjcr/ytae094

**Published:** 2024-02-20

**Authors:** Sakiko Gohbara, Yasuhide Mochizuki, Takanari Fujii, Hideshi Tomita, Toshiro Shinke

**Affiliations:** Division of Cardiology, Showa University School of Medicine, 1-5-8 Hatanodai, Shinagawa-ku, Tokyo 142-8555, Japan; Division of Cardiology, Showa University School of Medicine, 1-5-8 Hatanodai, Shinagawa-ku, Tokyo 142-8555, Japan; Pediatric Cardiology and Adult Congenital Heart Disease Center, Showa University Hospital, Tokyo, Japan; Pediatric Cardiology and Adult Congenital Heart Disease Center, Showa University Hospital, Tokyo, Japan; Division of Cardiology, Showa University School of Medicine, 1-5-8 Hatanodai, Shinagawa-ku, Tokyo 142-8555, Japan

**Keywords:** Isolated right ventricular hypoplasia, Cyanotic atrial septal defect, Balloon occlusion test, Case report

## Abstract

**Background:**

Hypoxaemia in isolated right ventricular (RV) hypoplasia (IRVH) is primarily caused by a right-to-left shunt (RLS) at the atrial level, such as an atrial septal defect (ASD). When considering closure of the RLS, it should be closed only after ensuring that it will not cause right-sided heart failure (HF).

**Case summary:**

A 21-year-old woman had been experiencing shortness of breath during exertion since childhood. Transthoracic and transoesophageal echocardiography revealed an ASD with bidirectional shunting, and microbubble test revealed a marked RLS. Cardiac magnetic resonance imaging revealed a hypoplastic RV end-diastolic volume corrected for body surface area of 47 mL/m^2^ (70% of normal range). Right heart catheterization revealed a decreased Qp/Qs ratio of 0.89 and a pressure waveform with a clear increase in the ‘A’-wave, although the mean right atrial pressure was not high (4 mmHg). Therefore, the patient was diagnosed with cyanotic ASD and IRVH. A temporary balloon occlusion test was performed to evaluate the right-sided heart response to capacitive loading prior to ASD closure. After treatment, the patient’s improved markedly. The pre-operative brain natriuretic peptide (BNP) level was normal; however, 6 months after ASD closure, the BNP level was elevated, and the continuous-wave Doppler waveform of pulmonary regurgitation at the time of transthoracic echocardiography changed, suggesting an increase in diastolic RV pressure.

**Discussion:**

When ASD is complicated by hypoxaemia, the possibility of IRVH, although rare, should be considered. Another difficult point is determining whether the ASD can be closed, considering its immature RV compliance.

**Learning points**

- The combination of structural heart disease and hypoxia should trigger for the evaluation for a right-to-left shunt.
- The combination of right ventricular hypoplasia and an atrial septal defect (ASD) is challenging from a clinical management perspective and necessitates thorough haemodynamic evaluation prior to closure of the ASD.

## Introduction

Isolated right ventricular (RV) hypoplasia (IRVH) is a rare congenital heart disease frequently associated with shunting at the atrial level, resulting in a right-to-left shunt (RLS) due to a rapid increase in RV diastolic pressure.^1,2^ The resulting hypoxaemia from early childhood often leads to a diagnosis. To avoid increasing pressure in the venous system, including the right atrium (RA), it is important to ensure that the pressure levels are stable before closing an atrial septal defect (ASD) with cyanosis. However, the degree of IRVH varies among patients, and currently, no criteria exist regarding closure.

## Summary figure

**Table:** 

Date	Events
From childhood to 21 years of age	The patient has always been aware of shortness of breath even during mild exertion.
May 2022	She visited her previous physician with worsening cough and dyspnoea, being hypoxaemic with saturation of percutaneous oxygen <90% in room air.
	Transthoracic echocardiography with microbubble test.
	Contrast-enhanced computed tomography.
Early June 2022	Transoesophageal echocardiography revealed right-to-left shunt (RLS) with atrial septal defect (ASD).
	Cardiac magnetic resonance imaging.
	Right heart catheterization.
Late June 2022	Percutaneous ASD closure was successfully performed.
July 2022	Transthoracic echocardiography with microbubble test 1 month post-operatively showed residual Grade 4 RLS.
March 2023	Saturation of percutaneous oxygen at rest was maintained at a normal level in room air. Transthoracic echocardiography was performed 9 months after ASD closure. The atrial septum appeared to be fixed with a closure device, and colour Doppler did not reveal any residual shunting.

## Case presentation

The patient in this case was a 21-year-old woman diagnosed with a ventricular septal defect (VSD) soon after birth. The VSD closed spontaneously at 3 years and 6 months of age, and she was discharged from follow-up.

**Figure 1:**
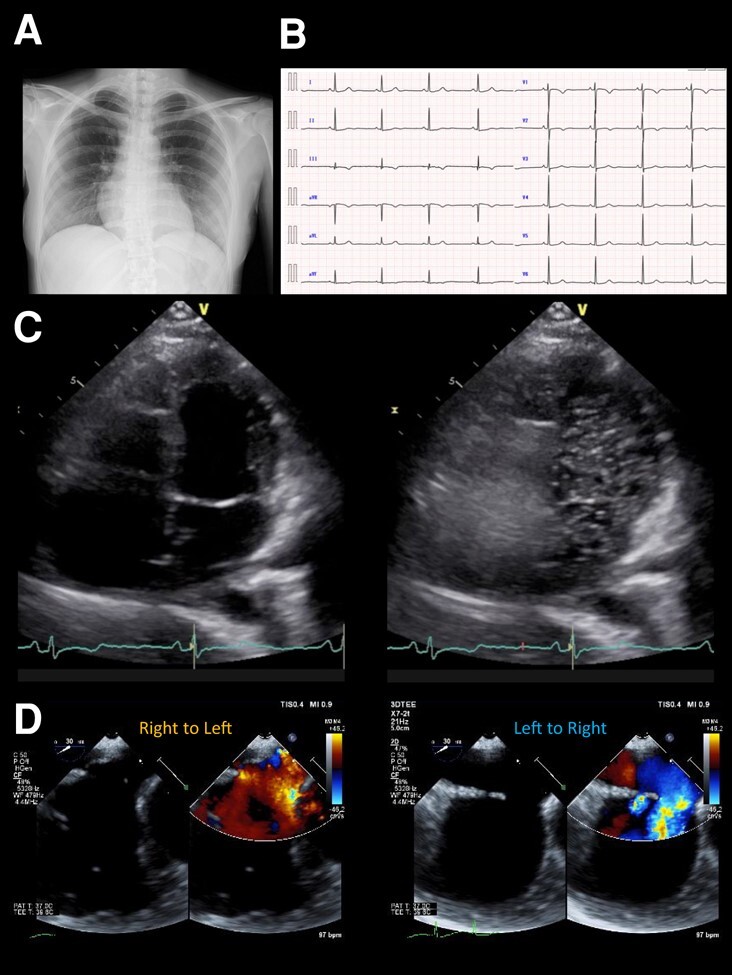
(*A*) Chest X-ray photograph showing a slightly enlarged cardio-thoracic ratio of 56%. (*B*). Electrocardiogram on admission showing a normal sinus rhythm of 53 b.p.m. without any bundle branch blocks. (*C*) Transthoracic echocardiography at rest showing a Grade 4 right-to-left shunt at the atrial level. (*D*) Colour Doppler flow imaging of transoesophageal echocardiography showing a bidirectional shunt through the atrial septal defect.

**Figure 2:**
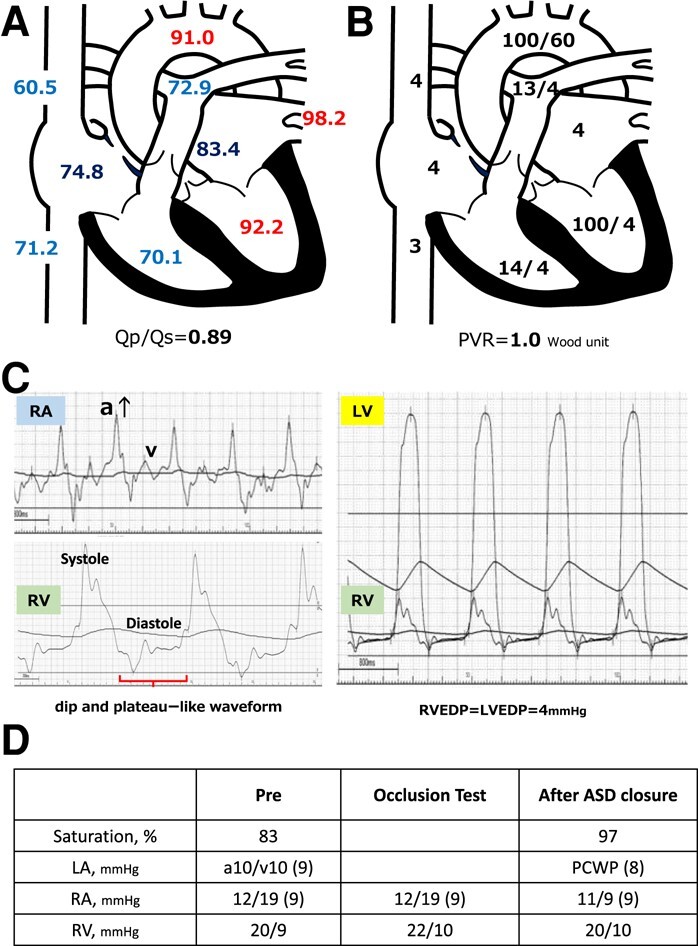
(*A*) Presenting oxygen saturation (%) under 3 L/min of oxygen administered by mask. Oxygen saturation increased in the right atrium and decreased from the pulmonary vein to the left atrium. (*B*) Pressure (mmHg) in each lumen of the right heart catheter is shown immediately after hospitalization. Each atrium and vein presents the mean pressure, whereas the ventricles, aorta, and pulmonary artery show systolic/diastolic pressure data. PVR, pulmonary vascular resistance. (*C*) The pressure waveforms by right heart catheterization showed a significant increase of ‘A’-wave in the right atrial pressure wave and dip-and-plateau pattern in the right ventricle. RA. right atrium; RV, right ventricle; LV, left ventricle; EDP, end-diastolic pressure. (*D*) Right heart catheter at ASD closure after volume loading with 500 mL normal saline shows oxygen saturation increases without any increase in central venous pressure or right atrial or ventricular pressure. PCWP, pulmonary capillary wedge pressure; ASD, atrial septal defect.

**Figure 3:**
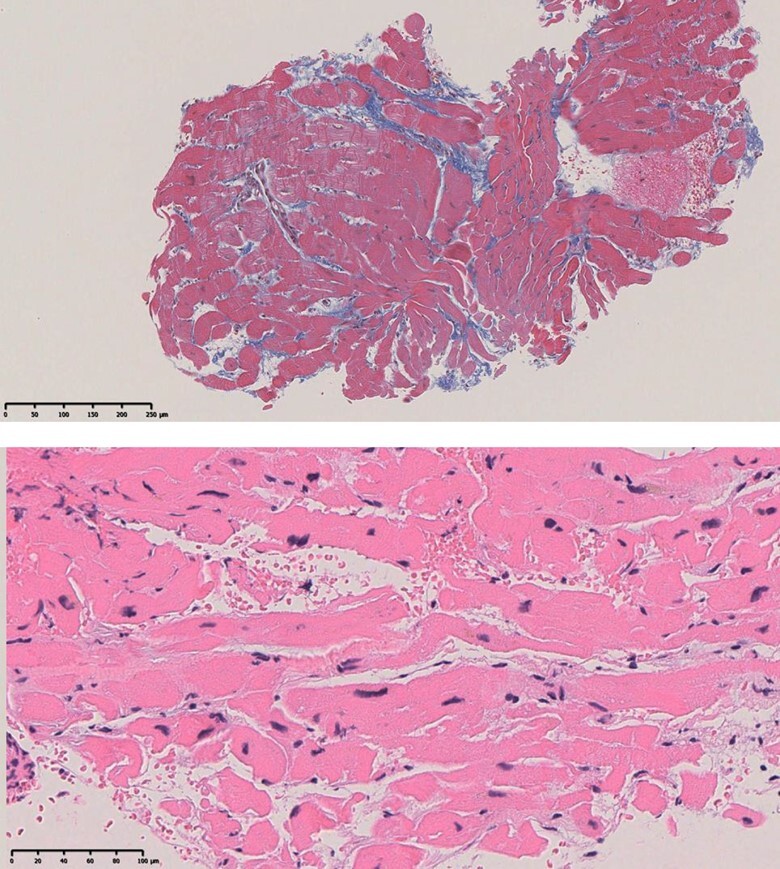
The upper panel is a weakly magnified Masson stain (×40) showing mild stromal fibrosis. The lower panel is a strongly magnified (×200) haematoxylin eosin-stained section showing a marked discrepancy in cardiomyocyte diameter.

In May 2022, the patient visited a clinic with a chief complaint of worsening dyspnoea and presented with severe hypoxaemia. Contrast-enhanced computed tomography revealed an ASD. The patient was transferred to our hospital to determine whether the ASD with RLS should be closed. Physical examination showed an SpO_2_ of 88% in the room air, blood pressure of 96/64 mmHg, and a regular pulse of 60 b.p.m. There were no significant heart murmurs, split heart sound, or specific findings in the jugular vein. Laboratory findings demonstrated haemoglobin and haematocrit levels were relatively high at 14.1 g/dL (normal range: 12–16g/dL) and 42.8% (normal range: 35–45%) for a young woman with menorrhagia, respectively, and no increase in brain natriuretic peptide (BNP) level to 12.3 pg/mL (normal range: <18.4pg/mL). Chest radiography revealed mild cardiomegaly, with no abnormal findings in the lung field (*Figure 1A*). The 12-lead electrocardiogram showed normal sinus rhythm without any conduction abnormalities (*Figure 1B*). V1 and V2 showed prominent R- and minor negative T-waves but no P-wave augmentation indicative of increased RA load. Transthoracic echocardiography (TTE) revealed a mildly reduced left ventricular (LV) ejection fraction of 47%, with LV end-diastolic and end-systolic volumes of 78 and 41 mL, respectively (Supplementary material online, *Movie S1*). Atrial septal defect with bidirectional shunting was visible, and TTE with microbubble test revealed a Grade 4 RLS at the atrial level, even without the Valsalva manoeuver (*Figure 1C*, Supplementary material online, *Movie S2*). Basal and mid-cavity RV linear dimension at end-diastole in the RV-focused view were 35 and 30 mm, respectively. Similarly, transoesophageal echocardiography showed a bidirectional shunt on colour Doppler, with an 11 mm secundum ASD located anterosuperiorly (*Figure 1D*, Supplementary material online, *Movie S3*). On cardiac magnetic resonance imaging (cMRI), the indexed RV end-diastolic volume was 47 mL/m^2^ (female normal range: 67 ± 10), indicating borderline RV hypoplasia.^3^ The ratio of LV to RV at end-diastolic volume was 1.4, indicating no RV enlargement despite the ASD. No late gadolinium enhancement was identified in the RV wall. An abnormal inflow from the right to the left atrium (LA) was observed (Supplementary material online, *Movie S4*). Right heart catheterization performed at the time of admission revealed a decreased Qp/Qs ratio of 0.89 with reduced oxygen saturations in the LA sample compared with the pulmonary venous sample, indicating right-to-left shunting at the level of the atria. A step-down of oxygen saturation from the RA to the RV reflected the inflow from the inferior and superior vena cava, while a step-up from the LA to the LV reflected the inflow from the pulmonary veins. Pulmonary hypertension was ruled out, as pulmonary arterial pressure was within normal limits at 13/4 (8) mmHg and pulmonary vascular resistance was low at 1.0 Wood unit (*Figure 2A* and *B*). The pressure waveform showed a giant ‘A’-wave, even though the mean RA pressure was not high, at 4 mmHg. The RV pressure waveform showed like a dip-and-plateau pattern, suggesting a diminished filling capacity. The simultaneous LV–RV pressure waveforms showed little difference between the two ventricles, and the RV end-diastolic pressure (RVEDP) was relatively high, suggesting RV diastolic dysfunction but was considered mild (*Figure 2C*). Right ventriculography revealed a relatively hypoplastic RV apex, an enlarged RA, and contrast flow from the RA to the LA (Supplementary material online, *Movie S5*). Myocardial biopsy was performed at the RV septum to exclude specific cardiomyopathy with identifiable cause. The RV endomyocardial biopsy showed evidence of increased fibrosis at a young age, size irregularities, as well as myocyte atrophy and hypertrophy, particularly around the interstitial fibrosis (*Figure 3*). In quantitative assessment, 74 cardiomyocyte diameters were measured from 7 different RV biopsy specimens. The mean RV myocyte diameter was 16.2 µm, the maximum 28.4 µm, and the minimum 6.3 µm (reference value <15 µm). Because of the relatively large standard deviation of 5.2 µm, the RV cardiomyocytes were identified as irregular in size. These findings led us to conclude that her severe hypoxaemia was due to a bidirectional shunt via an ASD, complicated by IRVH.

**Figure 4:**
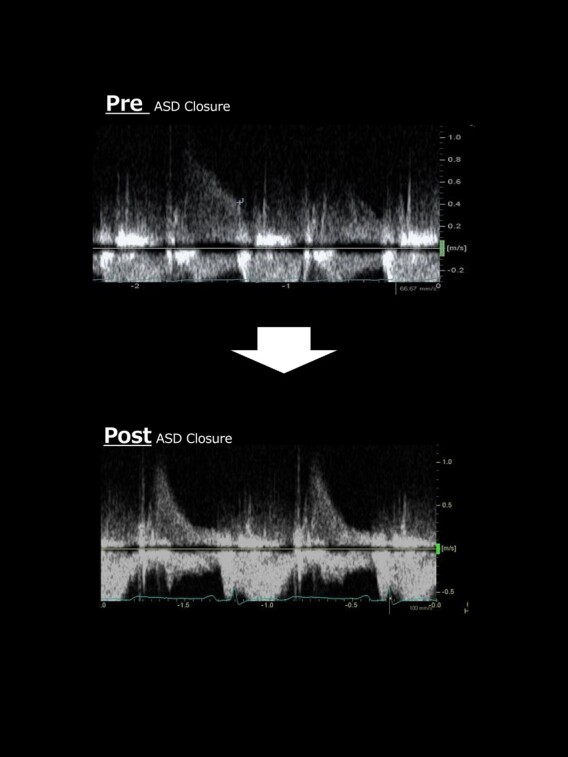
The change of continuous-wave Doppler waveform of the pulmonary regurgitation between before and after the atrial septal defect closure shows a steep decrease in pressure gradient suggesting an increase in diastolic right ventricular pressure.

Prior to transcatheter ASD closure, a balloon occlusion test was performed while intraoperative volume loading with 500 mL normal saline. The occlusion test proved that ASD closure was feasible, as the patient was kept for 10 min with the shunt completely occluded and oxygen saturation increased without any increase in central venous pressure or RA pressure (*Figure 2D*). ASD closure was then safely performed using the GORE® CARDIOFORM ASD Occluder 37 mm closure device. TTE with microbubble test 1 month post-operatively showed residual Grade 4 RLS. However, SpO_2_ remained the normal range in room air. Interestingly, the BNP level was increased to 153 pg/dL 6 months after ASD closure and then peaked. However, right heart failure (HF) symptoms such as pretibial oedema and pleural effusion did not occur throughout the post-operative course. After 1 year without diuretics, BNP level dropped to 85.6 pg/dL. TTE was performed 9 months after ASD closure. The atrial septum was fixed with a closure device, and colour Doppler did not reveal any residual shunting. The continuous-wave Doppler waveform of the pulmonary regurgitation showed a steep decrease in the pressure gradient, indicating an increase in diastolic RV pressure (*Figure 4*).

## Discussion

This case report describes a 21-year-old woman presenting with hypoxaemia. Of note in this case, despite a significant ASD, the RV was relatively hypoplastic with reduced compliance, which resulted in resistance to inflow from the RA to the RV, with ASD-mediated RLS causing hypoxaemia.

First, hypoxaemia associated with ASD requires consideration of several potential pathologies, including right heart system overload, congenital abnormalities, and anatomic abnormalities.^1,4^ Anatomical abnormalities may also be related to the inferior vena cava orientation and external cardiac factors, including the Eustachian valve and diaphragmatic hernia.^5^ With these conditions ruled out, it is reasonable to consider the pathogenesis of an ASD-mediated RLS complicated by IRVH. Isolated RV hypoplasia is an extremely rare disease with a genetic component reported in some cases.^2,6^ This case had no familial occurrence of congenital heart disease, including IRVH. While most cases of IRVH are reported to be associated with atrial shunts, clear diagnostic algorithms or clinical guidelines are currently not provided. In this case, VSD was detected at birth but closed spontaneously. By definition, IRVH is not affected by tricuspid or pulmonary valve morphology abnormalities or VSD,^1^ but VSD can complicate this condition.^7,8^

The interpretation of results from right heart catheterization is important because RV constrictive dysfunction can lead to hypoxaemia through the RLS. A dominant ‘A’-wave in the RA pressure waveform and a dip-and-plateau pattern in the RV waveform as observed in this case may reflect RV insufficient filling capacity.^9^ The timing of the augmented A-wave in RA and the respiratory changes in preload may cause the bidirectional shunt at the atrium septum. Independent prognostic factors include the onset of symptoms and diagnosis at <1 year of age, concomitant HF, and elevated RVEDP (>12 mmHg).^1^

It should be also emphasized that the impact of RLS closure on the right heart in patients with reduced RV compliance should be thoroughly tested prior to closure. In general, ASD closure in patients including IRVH and Ebstein anomaly requires a balloon occlusion test to estimate the increase in pressure in the right side heart after occlusion.^10^ Closure is reported to be possible if the mean RA pressure increases by no more than 20% during balloon occlusion test.^11^ In this case, the RVEDP was low and RA pressure did not increase during the occlusion test with volume loading; therefore, ASD closure was considered possible. However, BNP levels were transiently elevated post-operatively, and the pulmonary regurgitation waveform during the follow-up TTE indicated an increase in RV diastolic pressure, similar to that of constrictive pericarditis. This was a noteworthy event in the post-closure report. Additionally, we conducted an RV endocardial biopsy. Pathological examination revealed intramyocardial fibrosis, with mixed findings of myocyte hypertrophy and atrophy, which indicate a condition of impaired myocardium.^12^ Few reports exist on the myocardial pathology associated with IRVH. Previously, diffuse subendocardial or transmural late gadolinium enhancement of the RV on cMRI has been reported.^13^ Here, we report a case of transcatheter closure of a cyanotic ASD complicated by IRVH in an adult woman.

## Conclusion

This case report provides valuable data on the post-operative course and pathological findings of IRVH.

## Supplementary Material

ytae094_Supplementary_Data

## Data Availability

The data underlying this article will be shared on reasonable request to the corresponding author.

## Lead author biography

Dr Sakiko Gohbara graduated from Kawasaki Medical School in 2020. She is currently working in the Department of Cardiology, Showa University, in Tokyo, Japan, specializing in cardiac imaging, especially in valvular heart disease, cardiomyopathy, and congenital heart disease.
